# Single-Center Experience of Control of Ventilator-Circuit-Transmitted *Burkholderia cepacia* Outbreak in an Intensive Care Unit

**DOI:** 10.3390/tropicalmed8070335

**Published:** 2023-06-23

**Authors:** Bing-Jie Shen, Jann-Tay Wang, Hou-Tai Chang, Shan-Chwen Chang, Chun-Hsing Liao

**Affiliations:** 1 Department of Radiation Oncology, Fu Jen Catholic University Hospital, Fu Jen Catholic University, New Taipei City 24352, Taiwan; bjshen@ntu.edu.tw; 2 School of Medicine, College of Medicine, Fu Jen Catholic University, New Taipei City 24205, Taiwan; 3 Division of Infectious Disease, Department of Internal Medicine, National Taiwan University Hospital, Taipei 100225, Taiwan; wangjt1124@ntu.edu.tw; 4 Department of Critical Care Medicine, Far Eastern Memorial Hospital, New Taipei City 22060, Taiwan; houtai38@gmail.com; 5 Division of Infectious Disease, Department of Internal Medicine, Far Eastern Memorial Hospital, New Taipei City 22060, Taiwan; 6 School of Medicine, National Yang Ming Chiao Tung University, Taipei 100147, Taiwan

**Keywords:** outbreak, device-related infection, surveillance culture

## Abstract

*Burkholderia cepacia* is an emerging nosocomial pathogen frequently associated with outbreaks, but the exact transmission route of this pathogen can at times be elusive in spite of extensive environmental investigative cultures. Active surveillance for sputum cultures was performed for all patients from September 2008 to September 2009 in an intensive care unit (ICU) with *B. cepacia* outbreak. With evidence of persistent positive conversion of sputum cultures (colonization) and infections among patients, discontinuing re-usable ventilator circuits was introduced. A total of 689 patients were admitted to this unit for a mean duration of 8.7 ± 7.5 days. There were 489 patients (71.0%) with a stay for one to ten days; 161 (23.4%) patients for 11 to 20 days; and 39 (5.7%) with over 20 days. In the first group, 13.5% of patients had cultures converting from negative to positive, in contrast to 66.7% in the last group (*p* < 0.01). With intervention of using disposable ventilator circuits since June 2009, the incidence of isolated *B. cepacia* decreased gradually. The estimated 30-day isolation-free probabilities of the groups before, during, one month (August 2009) after, and two months (September 2009) after this intervention were 38.5%, 47.3%, 66.5%, and 96.0%, respectively (*p* < 0.01). Furthermore, the effect of discontinuing reusable ventilator circuit persisted in the following 6 years; both total isolates of *B. cepacia* and the infection caused by it were much lower compared to the outbreak period. In summary, this six-year outbreak in a medical ICU persisted until reusable ventilator circuits were discontinued in 2009. The effect of disposable circuits on the decreased incidence of *B. cepacia* infection maintained in the following years.

## 1. Background

*Burkholderia cepacia* has been recognized as an important pathogen in patients with cystic fibrosis for decades [1]. It is an emerging nosocomial pathogen that causes outbreaks via different sources [2,3,4]. Patients with an indwelling invasive device or intensive care unit (ICU) stay are at risk of acquiring *B. cepacia* in the hospital [4,5,6], but the precise mechanisms of an outbreak can be difficult to pinpoint and eliminate [7,8,9]. Two recent reviews illustrated that prolonged outbreaks were not infrequent, and emphasized the importance of medical product contamination [7,8].

Since January 2004, a protracted outbreak of patients with *B. cepacia* bacteremia was noted in an ICU of a Taiwanese hospital (five cases in the first 2 months in 2004 compared to three cases in year 2003). The initial recognition of the outbreak occurred after determination that over ninety percent of total *B. cepacia* isolated in the hospital central laboratory were from this specific unit. Pulse-field electrophoresis in the early phase showed 67% of the isolates could be considered a single clone [10]. Repeated environmental surveillance cultures were performed but no obvious transmission route was found. Environmental cleaning was enhanced and a checklist was implemented to decrease central catheter use combined with restriction of imipenem prescription. Compartmentalization with walls between patient beds was also constructed in 2006. However, sporadic cases of bacteremia were still found. Contact isolation for all patients with *B. cepacia* colonization was implemented in this unit in 2007. The number of bacteremia cases decreased, but patients continued to be colonized or infected with *B. cepacia* intermittently.

To delineate the occurrence and prevalence of *B. cepacia* transmission among patients within this ICU, surveillance sputum cultures for all patients admitted to this unit was initiated in September 2008 (at admission and every 3 days thereafter) as an enhanced infection control policy. We were surprised to find that in spite of the reduced incidence of bacteremia, the overall colonization rate of *B. cepacia* among patients in this unit remained high (30–40%). Positive sputum conversion rates were proportionate to length of ICU stay. Failure of sterilization for reusable ventilator circuit was suspected to be the culprit. In June 2009, we started to discontinue the use of reusable ventilator circuits, including tubes and humidifiers, and finally the conversion rate dropped markedly and few new cases occurred afterwards. Herein, we demonstrate the analysis of surveillance cultures and the process of controlling a protracted *B. cepacia* outbreak. Moreover, we monitor the occurrence of *B. cepacia* infection in the following years to see whether the effect of disposable circuit is persistent.

## 2. Materials and Methods

### 2.1. Hospital Setting and Identification of B. cepacia

Far Eastern Memorial Hospital (FEMH) is a 1040-bed hospital located in northern Taiwan. There are three adult ICUs, including medical ICU, surgical ICU, and cardiovascular ICU. The outbreak was detected in the medical ICU, which has 22 beds in the unit [10]. The ICU is divided into two adjacent chambers: an anterior room and a posterior room. This unit is responsible for patients with acute medical illness which requires intensive care, mainly respiratory failure, severe sepsis, multiple organ failure, etc. The surveillance of nosocomial infections at FEMH has been performed by the Nosocomial Infection Control Committee since 1985. All microbiology samples were processed in the central laboratory. *B. cepacia* was identified by colony morphology and biochemically with conventional tests (positive for lactose, maltose and sucrose, but negative for urease and lysine reaction). These isolates were further confirmed to be *B. cepacia* using PCR-restriction fragment length polymorphism analysis of the 16S rDNA as previously described [10,11,12].

### 2.2. Active Surveillance and Discontinuation of Reusable Ventilator Circuits

Due to the persistence of *B. cepacia* in this unit, active surveillance among all patients in the unit was performed as an advanced infection control policy. Sputum/lung aspirate (intubated patients) cultures were performed for all patients newly admitted to this ICU within 24 h of admission and routine surveillance cultures every 3 days thereafter and on the day that the patient was discharged from the ICU [13]. All cultures were sent to the central laboratory within 6 h for bacterial culture and subsequent microbiological studies. Patients positive for *B. cepacia* were put on contact isolation as per unit policy. Data on patient profiles, date of admission to ICU, bed and duration of stay in ICU, and results of cultures for each patient at three-day intervals were collected.

The results of sputum culture for each patient were summarized into three patterns: (1) All negative results through the course of stay in ICU; (2) All positive results through the course of stay in ICU; (3) Converting from negative results of the serial cultures to positive during the course of stay in ICU.

We divided the analysis into three stages: (1) Pre-intervention stage: reusable ventilator circuits were in use for all patients from September 2008 to May 2009; (2) Partial intervention stage: reusable ventilator circuits were only applied to half of the ICU patients from June 2009 to July 2009; (3) Complete intervention stage: reusable ventilator circuits were universally replaced with disposable circuits from August 2009 to September 2009.

### 2.3. Monitoring Infective State of Neighboring Beds (Spatial Effect)

The neighboring beds were potentially infective sources spatially associated with direct or indirect contact transmission. A neighboring bed with a positive patient staying on it for a certain period was considered an infective bed. The nearest one plays the most effective role of contact transmission.

We estimated of the risk of *B. cepacia* transmission from a neighboring bed with an infective patient staying on it just one-bed distance away during the same month. To clarify, the temporal and spatial association, as well as the odds ratios of *B. cepacia* colonization with exposure to an infective neighboring bed were stratified by the duration of the patients staying in the ICU.

### 2.4. Yearly Isolate Numbers of B. cepacia from Clinical Samples (Surveillance Cultures Not Included) and Nosocomial Infection Caused by B. cepacia

A central lab handles all clinical samples (surveillance culture is processed separately and not included in the database of clinical samples). Yearly isolate number of *B. cepacia* was retrieved from the hospital database. Isolate numbers in 2003 and 2004 were lower due to the fact that data collection was incomplete before awareness of the outbreak and there were no data for *B. cepacia* isolate in 2002. The identification method for *B. cepacia* did not change until MALDI-TOF was introduced for bacteria identification in late 2017. Yearly numbers of nosocomial infection at FEMH were regularly monitored since 1985 according to the criteria provided by CDC, Taiwan. For nosocomial infection caused by *B. cepacia*, lower respiratory tract infection is most common, followed by bloodstream infection [10].

### 2.5. Statistical Analysis

In the statistical analysis, means and standard deviations were calculated as summaries of continuous variables. Comparisons were performed with the Mann–Whitney U method for continuous variables, and with Chi-square test or Fisher’s exact test for categorical variables. The survival curves for patients converting from negative sputum culture or not were depicted using the Kaplan–Meier method, and the log-rank test to test differences between independent groups. A *p* value of < 0.05 was considered statistically significant. Data were collected in a Microsoft Excel database (Microsoft Excel 2003; Microsoft Corporation, Seattle, WA, USA) and analyzed with SPSS software for Windows (Release 10.0; SPSS, Inc., Chicago, IL, USA). Tracing the direct and indirect contact among all the patients at each time point and each spatial connection was computed on a Linux system with the R programming language [14].

## 3. Results

### 3.1. Secular Change of the Outbreak

**Figure 1:**
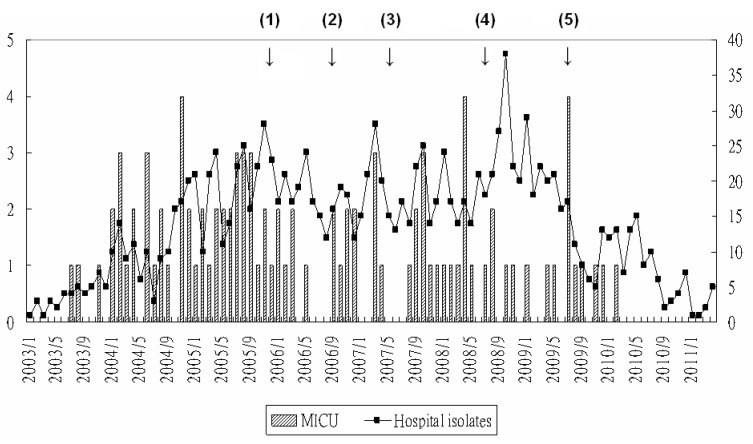
Secular change in nosocomial *Burkholderia cepacia* infections in a medical intensive care unit (MICU), contrast with total patient number colonized with *B. cepacia* in the hospital (culture results from active surveillance were not included). Interventions: (1) Environmental surveillance cultures and enhanced cleaning; (2) Improvement of MICU space and limitation of central catheter use; (3) Contact isolation of patients carrying *B. cepacia*; (4) Active surveillance for all MICU patients every three days; (5) Discontinuing reusable ventilator circuit.

The outbreak was first noted in early 2004. Five patients developed bacteremia within 2 months compared to three cases in 2003 (Figure 1). Over 90% of nosocomial *B. cepacia* infections occurred in this medical ICU, and pulse-field electrophoresis in the early phase showed 67% of the isolates could be considered as a single clone [10]. The monthly numbers of patients colonized with *B. cepacia* at our institute averaged twenty since 2005, in spite of multiple interventions, including contact isolation for all patients with *B. cepacia* colonization in 2007. Repeated and extensive environment surveillance cultures, including the water system, failed to isolate *B. cepacia* from the environment, except from ventilator tubing of patients colonized with *B. cepacia*. Some of the colonized patients developed *B. cepacia* infections subsequently, mostly pneumonia and bloodstream infection. Under such circumstances, active surveillance of sputum culture (every 3 days) for all patients admitted to this unit was started in September 2008 and ended in September 2009.

### 3.2. Characteristics of Patients and Results of Active Surveillance

The most common indication for admission to this ICU is respiratory failure, followed by severe sepsis (about 60–70%). The rate of patients under mechanical ventilation in this unit exceeds 90% regularly [10]. From September 2008 to September 2009, over the 13-month period, a total of 689 patients were admitted to this ICU for a mean duration of 8.7 ± 7.5 days, with mean durations of positive sputum culture per patient of 2.7 ± 6.0 days and negative sputum culture per patient of 5.9 ± 5.5 days.

There were 489 patients (71.0%) with an ICU stay for 1 to 10 days; 161 (23.4%) patients for 11 to 20 days; and 39 (5.7%) for more than 20 days (Table 1). In the subgroup with short stays lasting one to ten days, most patients (78.8%) had negative sputum cultures throughout. However, in the subgroup with stays exceeding 20 days, the majority (66.7%) had serial sputum cultures converting from negative to positive. There were no significant differences in the distribution of patients in the anterior and posterior rooms of the ICU.

During the period of reusable ventilator circuits from September 2008 to May 2009, the baseline rate of sputum culture converting from negative to positive was 27.5% in spite of previous interventions including enhanced environmental cleaning, limitation of central catheter use, and contact isolation of patients carrying *B. cepacia*. This culture conversion rate significantly decreased to 22.6% and 5.8% under intervention of discontinuing reusable ventilator circuits for half and all patients, from June 2009 to July 2009 and from August 2009 to September 2009, respectively. Only three patients developed *B. cepacia* infection in the following months. The monthly numbers of patients colonized with *B. cepacia* also waned with time (Figure 1).

### 3.3. Intervention of Discontinuing Reusable Ventilator Circuits

During the partial intervention stage, reuse of ventilator circuits was discontinued in half of 106 patients (Table 2). In the complete intervention stage, all 104 patients received disposable “use once only” ventilator circuits. There were no significant differences in the duration of stay nor chamber of stay among these groups.

### 3.4. Effects of Infective State of Neighboring Beds

During the outbreak of *B. cepacia*, direct or indirect contact transmissions could potentially occur among the infective and the susceptible patients (Table 3). The risks of *B. cepacia* transmission were significantly increased in those having a neighboring bed with a positive patient on it. The odds ratios were 2.90 in the group of the shortest duration of stay (1 to 10 days), 2.59 in the group of the intermediate duration of stay (11 to 20 days), and 12.39 in the group of the longest duration of stay (21 to 30 days). Prolonged duration (more than 20 days) of exposure to an infective neighboring bed had higher risk of direct or indirect contact transmission.

### 3.5. Effects of Discontinuing Reusable Ventilator Circuits

**Figure 2:**
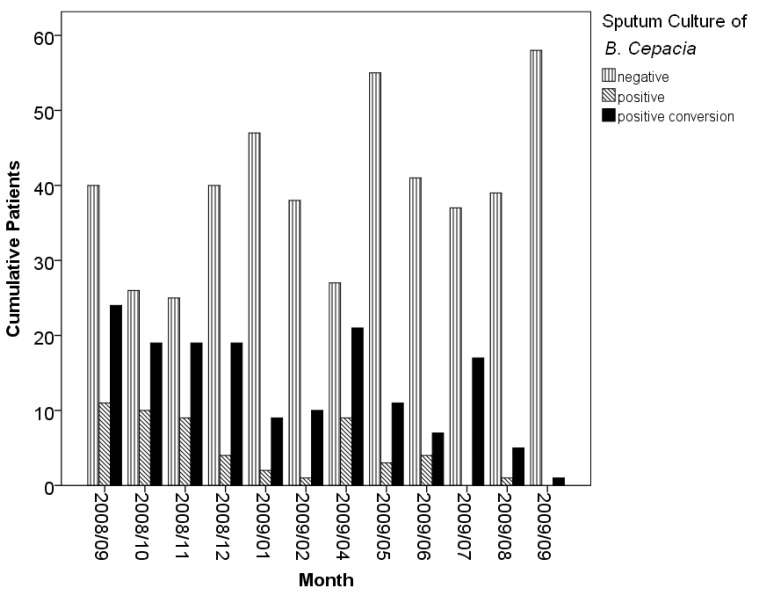
Cumulative results of *B. cepacia* sputum culture at three-day interval for each patient. With intervention of discontinuing reusable ventilator circuits since June 2009, the incidence of positive *B. cepacia* culture decreased gradually (*p* < 0.01).

**Figure 3:**
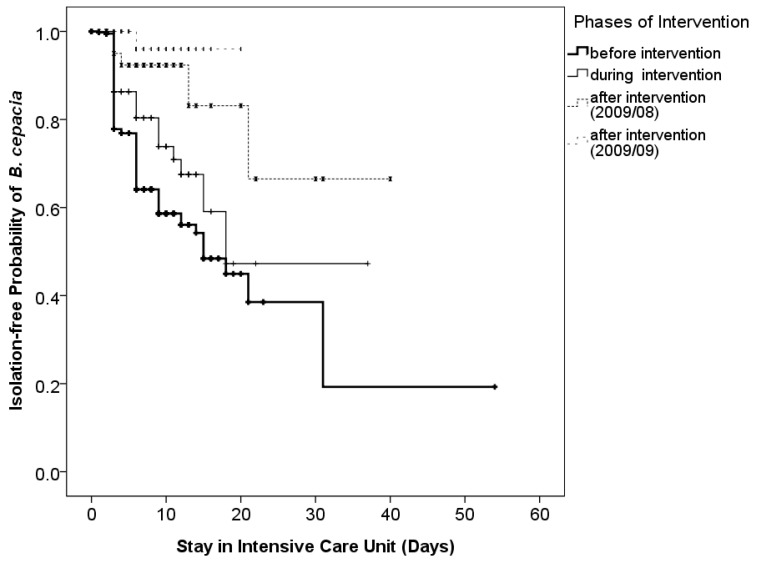
Intervention of discontinuing all reusable ventilator circuits resulted in significantly decreased positive culture rate. The estimated 30-day culture-free probabilities of the pre-intervention stage (before intervention), the partial intervention stage (during intervention), the complete intervention stage (August 2009), and the complete intervention stage (September 2009) are 38.5%, 47.3%, 66.5%, and 96.0%, respectively (*p* < 0.01).

Discontinuing reusable ventilator circuits was the most influential factor identified in this study (Figure 2). With intervention of discontinuing reusable ventilator circuits since June 2009, the incidence of isolated *B. cepacia* declined. Furthermore, prolonged latency of transmission under the intervention was observed (Figure 3). The estimated 30-day isolation-free probabilities of the groups before, during, one month (August 2009) after, and two months (September 2009) after the intervention were 38.5%, 47.3%, 66.5%, and 96.0%, respectively (*p* < 0.01).

### 3.6. Follow-Up of Total Isolates of B. cepacia and Infection Caused by B. cepacia at FEMH

**Figure 4:**
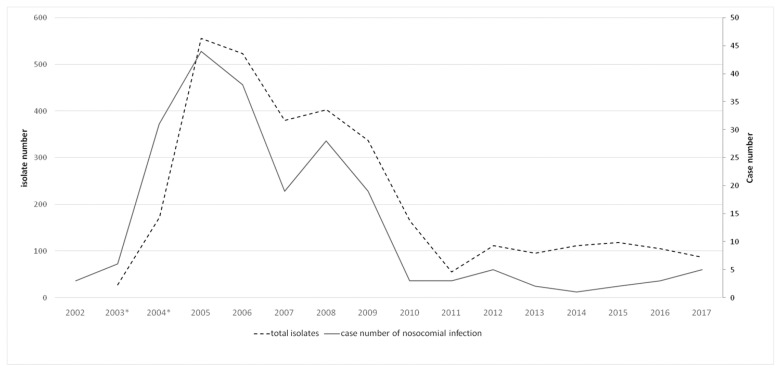
Six-year follow-up after the control of outbreak. Case numbers of nosocomial infection caused by *B. cepacia* and total isolate numbers of *B. cepacia* at FEMH. * Isolate numbers in 2003 and 2004 were lower due to the fact that data collection were incomplete before awareness of the outbreak and there were no data for 2002.

After the intervention and its significant result, all reusable ventilator circuits were discontinued at FEMH. The yearly number of total isolates of *B. cepacia* were around 100 and nosocomial infection caused by *B. cepacia* was below five cases since 2010 (Figure 4). The effect persisted for more than 6 years.

## 4. Discussion

This study describes the eventually successful management of a six-year outbreak of *Burkholderia cepacia* in an ICU in Taiwan. The outbreak was not controlled until application of active surveillance to demonstrate the high sputum culture conversion rates among patients admitted to this unit in spite of prior interventions, including contact isolation for patients carrying *B. cepacia*. Adoption of disposable ventilator circuits, including tubes, humidifiers, and filters in this unit stopped the positive sputum conversion (colonization) and prevented subsequent infections in the following years.

Outbreaks secondary to *B. cepacia* are not rare. Common sources related to *B. cepacia* outbreak include contaminated chlorhexidine [15], albuterol [3,16], intravenous bromopride [17], multi-dosed medications [18,19], alcohol-free mouth wash [20], table cloth [21], and respiratory therapy device [22]. However, it is not uncommon for the transmission route to be elusive [7,8,9,23,24]. In the early phase of this outbreak, we attempted to identify a single cause to explain the outbreak. In spite of repeated environmental surveys and reviews of medications used by patients, we failed to determine the transmission route. Thus, several generalized interventions including decreasing catheter use and contact isolation of colonized patients were implemented. However, the outbreak persisted. Furthermore, the prolonged and widespread nature of infections lowered the possibility of medications or disinfectant-related outbreak. To decrease the possibility of transmission due to medical staff, we even built walls between ICU beds, though all the cultures from staff hands were never positive for *B. cepacia*.

Active surveillance is most typically part of infection control policies for methicillin-resistant *Staphylococcus aureus* and for vancomycin-resistant *Enterococcus* [25]. There have been fewer reports on the application of active surveillance to control outbreaks due to Gram-negative bacteria such as carbapenem resistance *K. pneumoniae* [26] and *A. baumannii* outbreak [27]. In this study, we actively surveyed all patients admitted to this unit over one year and successfully demonstrated that the transmission of *B. cepacia* in this unit was more related to length of stay than proximity to a colonized patient source. From the risks imparted with time, we inferred that failure to disinfect reusable equipment such as mechanical ventilator circuits could play a key role in perpetuating this outbreak.

Thermal sensors of ventilators are infamous culprits of *B. cepacia* outbreaks in several reports [28,29,30]. Accordingly, we disinfected our thermal sensors and flow sensors with ethylene oxide in the early months of active surveillance. However, *B. cepacia* colonization was also found in patients using ventilators without thermal sensors (Bird 8400), and the application of ethylene oxide did not stop the outbreak. Major ventilators in this unit include EVITA 2-dura, Evita 4, and Bird 8400, and the chance for colonization of *B. cepacia* among patients was not different among ventilators.

We then postulated that the ventilator circuits could be the culprit source based on three observations: first, 99% of patients with *B. cepacia* infections were on ventilator support [10]; second, the only site that *B. cepacia* was repeatedly isolated was from the circuits. Third, each unit managed its ventilator circuit, and the outbreak was mainly confined to the medical ICU. In the report of Hartstein et al. of the *Acinetobacter* outbreak due to ventilator circuits, it was found that barrier precautions and improved staff handwashing did not diminish the frequency of new cases [31], and 18% of reusable ventilator circuits and resuscitation bags were culture positive for the outbreak clone after pasteurization. They adopted terminal ethylene oxide sterilization of these devices which terminated their outbreak. At our institute, adopting full-scale ethylene oxide sterilization for all the circuits was not feasible. Thus, we started to use disposable circuits. At first, only half of the patients used disposable circuits, and the positive conversion rates did not decline significantly. In August 2009, we discontinued all reusable ventilator circuits, and within 2 months, the positive conversion rates dropped markedly.

There is limited information about sterilization of ventilator circuits in the literature [32], and practices vary among institutes. At our hospital, the cleaning system T-840 (HAMO, Switzerland) is used, with a protocol as follows: pre-wash for 5 min with a 45 °C water, wash for 1.5 min with a 60 °C water and detergent (HAMO criti-klenz), rinse for 2.5 min with a 65 °C water, final wash with a 85 °C reverse osmosis water for 6.5 min, and dry with a 85 °C air for 70 s. *B. cepacia* were frequently positive from samples collected from the circuits (including tubes, humidifier, water traps, and even outflow filter) of colonized patients in our surveillance cultures [33,34]. On the contrary, *B. cepacia* was only cultured from the environment once on the surface of a trash can, despite strenuous and extensive environmental surveys. Initially, intrinsic contamination from patients was considered, and surveillance cultures from circuits became negative after the pasteurization system. We even randomly selected 20 tubes after disinfection and cut the tubes open for complete sampling, but the culture remained negative. Judging from the dramatic response after adoption of universal disposable circuits, we believe that contaminated ventilator circuit was the root cause of this six-year *B. cepacia* outbreak. The possible explanations for our failure to culture *B. cepacia* from the circuits include sampling inadequacies and culture method limitations in detecting very small amounts of *B. cepacia*.

The water system at FEMH was studied one year later after the outbreak was controlled due to one patient becoming diagnosed with nosocomial legionellosis [35]. *Pseudomonas* spp., *Stenotrophomonas* spp., and *Sphingomonas* spp. were the major organisms isolated from the water system, not *B. cepacia*. The long-term effect of single-use ventilator circuit was demonstrated by few nosocomial infections caused by *B. cepacia* and markedly decreased total isolate numbers in the following years. However, the overall nosocomial infection rate at this unit did not change significantly in spite of disappearance of *B. cepacia* [35]. 

## 5. Conclusions

In summary, we present the process of managing a six-year *B. cepacia* outbreak. Using extensive active surveillance, we demonstrate that transmission of *B. cepacia* is device and time related. There are growing numbers of new devices with different components, and the importance of disinfecting each component properly to minimize the possibility of iatrogenic transmission cannot be overstated. In circumstances when this is not possible, replacing reusable parts with disposable parts circumvents this barrier to pathogen elimination.

## Figures and Tables

**Table 1:** Characteristics of 689 patients receiving surveillance sputum culture for *B. cepacia*.

Variable	Results of Serial Sputum Cultures			Total (%)	*p* Value
	All Negative (%)	All Positive (%)	Converting to Positive (%)		
Duration of Stay (days)					
1–10	386 (78.8)	37 (7.6)	66 (13.5)	489 (100)	<0.01
11–20	76 (47.2)	15 (9.3)	70 (43.5)	161 (100)	
>20	11 (28.2)	2 (5.1)	26 (66.7)	39 (100)	
Date of Admission					
September 2008–May 2009	298 (60.3)	49 (10.2)	132 (27.5)	479 (100)	<0.01
June 2009–July 2009 *	78 (73.6)	4 (3.8)	24 (22.6)	106 (100)	
August 2009–September 2009 ^†^	97 (93.3)	1 (1.0)	6 (5.8)	104 (100)	
Chamber of Stay					
Anterior Room	253 (72.7)	23 (6.6)	72 (20.7)	348 (100)	0.07
Posterior Room	220 (64.5)	31 (9.1)	90 (26.4)	341 (100)	
Total	473 (68.7)	54 (7.8)	162 (23.5)	689 (100)	

* Half of patients used disposable ventilator circuits. ^†^ All patients used disposable ventilator circuits.

**Table 2:** Groups of 689 patients before, during, and after intervention.

Variable	Intervention Stages			Total (%)	*p* Value
	Pre-Intervention (%)	Partial Intervention (%)	Complete Intervention (%)		
Duration of Stay (days)					
1–10	342 (70.0)	71 (14.5)	76 (15.5)	489 (100)	0.84
11–20	111 (68.9)	27 (16.8)	23 (14.3)	161 (100)	
>20	26 (66.7)	8 (20.5)	5 (12.8)	39 (100)	
Chamber of Stay					
Anterior Room	246 (70.7)	53 (15.2)	49 (14.1)	348 (100)	0.73
Posterior Room	233 (68.3)	53 (15.5)	55 (16.1)	341 (100)	
Total	479 (69.5)	106 (15.4)	104 (15.1)	689 (100)	

**Table 3:** Risks of *B. cepacia* transmission from a neighboring bed with an infective patient during the same month.

Duration of Stay (Days)	*B. cepacia* (+)		*B. cepacia* (−)		Odds Ratio	*p* Value
	Neighboring Bed (+)	Neighboring Bed (−)	Neighboring Bed (+)	Neighboring Bed (−)		
1–10	88	15	258	128	2.90	<0.01
11–20	68	17	46	30	2.59	<0.01
21–30	19	1	4	3	12.39	0.04
Total	175	33	308	161		

## Data Availability

All data are available via mail to the corresponding author.
